# Biphasic Oxidation of Oxy-Hemoglobin in Bloodstains

**DOI:** 10.1371/journal.pone.0021845

**Published:** 2011-07-15

**Authors:** Rolf H. Bremmer, Daniel M. de Bruin, Maarten de Joode, Wybren Jan Buma, Ton G. van Leeuwen, Maurice C. G. Aalders

**Affiliations:** 1 Biomedical Engineering and Physics, Academic Medical Center, University of Amsterdam, Amsterdam, The Netherlands; 2 Van 't Hoff Institute for Molecular Sciences, University of Amsterdam, Amsterdam, The Netherlands; 3 Biomedical Photonic Imaging, MIRA Institute for Biomedical Technology and Technical Medicine, University of Twente, Enschede, The Netherlands; University of Milano-Bicocca, Italy

## Abstract

**Background:**

In forensic science, age determination of bloodstains can be crucial in reconstructing crimes. Upon exiting the body, bloodstains transit from bright red to dark brown, which is attributed to oxidation of oxy-hemoglobin (HbO_2_) to met-hemoglobin (met-Hb) and hemichrome (HC). The fractions of HbO_2_, met-Hb and HC in a bloodstain can be used for age determination of bloodstains. In this study, we further analyze the conversion of HbO_2_ to met-Hb and HC, and determine the effect of temperature and humidity on the conversion rates.

**Methodology:**

The fractions of HbO_2_, met-Hb and HC in a bloodstain, as determined by quantitative analysis of optical reflectance spectra (450–800 nm), were measured as function of age, temperature and humidity. Additionally, Optical Coherence Tomography around 1300 nm was used to confirm quantitative spectral analysis approach.

**Conclusions:**

The oxidation rate of HbO_2_ in bloodstains is biphasic. At first, the oxidation of HbO_2_ is rapid, but slows down after a few hours. These oxidation rates are strongly temperature dependent. However, the oxidation of HbO_2_ seems to be independent of humidity, whereas the transition of met-Hb into HC strongly depends on humidity. Knowledge of these decay rates is indispensable for translating laboratory results into forensic practice, and to enable bloodstain age determination on the crime scene.

## Introduction

Bloodstains at crime scenes have prominent forensic value. The blood spatter pattern can assist in reconstructing crime events and DNA-profiling may lead to the suspect's identity. Despite the fact that determination of the age of a bloodstain has been a forensic quest in the last decades, the potential of age determination has not been exploited up till now. Techniques ranging from Atomic Force Microscopy [1], Electron Paramagnetic Resonance [2], and RNA degradation [3] have been explored to fulfill this omission. All these approaches confirm that the physical and chemical properties of bloodstains change over time. However, no technique has yet shown the precision and reproducibility needed for age determination in forensic practice. Recently, we have demonstrated that non-contact reflectance spectroscopy is a suitable candidate for bloodstain age estimation [4].

Reflectance spectroscopy is a widely used technique to determine the optical properties of biological samples. Quantitative analysis of reflectance spectra requires knowledge of two parameters: (i) the absorption coefficients of the expected chromophores, and (ii) the path length the photons have travelled through the sample prior to detection. When both are known, Beer's law can be applied for determination of the concentration of the chromophores. Multiple chromophores can be determined when the reflectance is measured and analyzed over a large spectral range, in our case 450–800 nm. The spectral absorption coefficients of the three main chromophores in bloodstains, μ_a_(λ), with λ denoting the wavelength, are very well known [5], and cover a range from 0.1 to 30 mm^−1^. The second parameter, the path length of the detected photons, however, does not only depend on the geometry of the illumination and detection setup, but also on the chromophores present. The latter dependency complicates the analysis of the reflectance spectra. Yet, by defining a relation between the absorption and scattering properties of the sample and the photon path length, the chromophores and photon path length can be measured simultaneously [6], [7].

Quantitative analysis of the reflectance spectra by photon path length modeling so far included the assumption that the scattering properties of the reference material and the bloodstain are equal [6], [7]. Therefore, additional experiments to determine whether scattering of the bloodstain on cotton is similar to clean cotton fabric are needed, e.g. by optical coherence tomography (OCT) [8]. OCT is a high-resolution, cross-sectional imaging technique widely used for medical applications [9], [10]. Quantitative analysis of the OCT signal in depth allows the determination of the scattering properties of the tissue under study [11].

Hemoglobin is the oxygen transporting molecule and consists of four heme subunits: two α chains and two β chains. Hemoglobin is the main chromophore in blood, since hemoglobin makes up 97% of the blood's dry content. Inside a healthy human body hemoglobin is mainly present in two forms: one without oxygen, de-oxyhemoglobin (deoxy-Hb), and one saturated with oxygen, oxy-hemoglobin (HbO_2_). Only a small part (∼1%) of HbO_2_ is auto-oxidized into met-hemoglobin (met-Hb) [12], which is reduced back to Hb by the reductase protein cytochrome *b5* [13]. Upon blood exiting the body, hemoglobin saturates with oxygen in the ambient environment. Due to a decreasing availability of cytochrome *b5*, the transition of HbO_2_ into met-Hb will no longer be reversed. Once hemoglobin is auto-oxidized to met-Hb, it will denature to hemichrome (HC) [14], [15], as is shown in Eq (1):
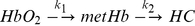
(1)The transition rate of HbO_2_ to met-Hb is represented by k_1_, and the transition rate of met-Hb to HC is represented by k_2_. Tsuruga *et al.* have shown that the autoxidation rate of aqueous HbO_2_, rate k_1_, can be described by first-order reaction kinetics [16]. The two oxidation rates of HbO_2_ rate have been attributed to fast oxidation of the α chains of hemoglobin and slow oxidation of the β chains. These oxidation rates have only been reported in aqueous solutions and not *in vivo* or in bloodstains. Because of the biphasic nature of the transition of HbO_2_ into met-Hb, k_1_ can be described as the sum of a fast rate and a slow rate, k_1_ = k_f_+k_s_. According to Tsuruga *et al.* [16] the oxidation of HbO_2_ is given by:
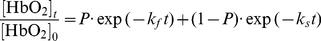
(2)In Eq (2). [HbO_2_]_t_ is the relative fraction of HbO_2_ in the bloodstain at time *t*, [HbO_2_]_0_ the relative fraction at *t* = 0, *k_f_* the fast rate constant, *k_s_* the slow rate constant, and *P* the fraction of the rapidly oxidizing hemoglobin.

The present study shows, to our best knowledge for the first time, oxidation decay rates of the subsequent steps in bloodstains and the dependency of these reaction rates on temperature and humidity. Until now, the ageing of bloodstains has been interpreted by empirical models [2], [17], [18], which hamper understanding of the oxidation decay rates and its dependency on temperature and humidity. We present reflectance spectroscopy measurements of bloodstains on cotton to determine the amount of hemoglobin derivatives in ageing bloodstains. Cross-sectional measurements of the bloodstain were recorded by optical coherence tomography to confirm the assumptions made for the quantitative reflectance approach. Validation of the method for chromophore concentration estimation was achieved by diluting whole blood with phosphate buffered saline and correlating the input dilution to the sum of the determined chromophores. Finally, bloodstains, stored under various environmental conditions were monitored for ten days. From the relative oxy-hemoglobin concentration biphasic oxidation rates were determined and measured as function of temperature and humidity.

## Methods

### 2.1 Ethics Statement

All research involving human subjects has been conducted on anonymized tissue. Informed written consent was obtained from each donor. The Internal Review Board of the Academic Medical Center Amsterdam was informed about design and purpose of this study. The IRB waived the need for approval, because of the non-clinical purpose of this study.

### 2.2 Bloodstains

Blood was drawn from a healthy nonsmoking volunteer. Three sets of samples were created. The first set was created by diluting whole blood with phosphate buffered saline to create eight stains on white cotton. All stains contained 50 µl of dilutions with varying blood volume fractions (BVF) ranging from 0 (no blood) to 1 (whole blood), with the following BVF: [0, 0.1, 0.2, 0.4, 0.6. 0.8, 1]. The bloodstains were measured three hours after deposition to allow the water in the bloodstain to evaporate.

The second set contained five times three bloodstains of 50 µl created on white cotton which were stored in a dark box at a temperature T = [−0°C, 4°C, 22°C, 29°C, 37°C] and at a humidity of 45±5%. During the first day the reflectance of bloodstains was measured every hour, thereafter daily for 10 days.

The third set contained three times three bloodstains of 50 µl created on white cotton which were stored in a dark box at T = 37±1°C at varying humidity of [20%, 50%, 70%]. During the first day the reflectance of bloodstains was measured every hour, thereafter daily for 8 days.

### 2.3 Optical Coherence Tomography

The OCT measurements were taken around 1300 nm where the absorption of the hemoglobin derivatives is minimal. By fitting the attenuated OCT signal to Beer's law, the attenuation coefficient can be determined. The cotton, with and without bloodstains, was probed and visualized with a commercially available 50 kHz swept source OCT system (Santec HSL 2000, 1300 nm center wavelength, 10 µm axial resolution, 11 µm lateral resolution). No additional image enhancement was performed on the presented image.

Using Beer's law, the detector current *i_d_* of the system is described as *i_d_*∝[exp(−2μ_t_z)]^1/2^ where μ_t_ is the attenuation coefficient and *z* is the depth of the light in the sample. The square root accounts for the fact that the detector current is proportional to the field rather than the intensity. The attenuation coefficient is then extracted from the OCT data by fitting Beer's law to the averaged A-scans from a selected region of interest in the OCT image (∼1000 A-scans of 4096 points, covering 1.5 mm scan length) [19]. To account for surface roughness of the cotton, all A-scans are aligned to straighten the transition between air and cotton. The fit model features three parameters; an amplitude for scaling signal intensity, the attenuation coefficient μ_t_, and a measure for the scattering of the cotton. The offset is fixed at the mean noise level.

### 2.4 Non-contact reflectance spectroscopy

Quantitative determination of the chromophore fraction requires two reflectance measurements: one of the bloodstain and one of the host material. The latter reference measurement is needed to correct for the scattering of the host material, lamp spectral calibration and system throughput. The calibration procedure and analysis of the reflectance spectra have been described in detail elsewhere [7].

The attenuation of the reflected light from the bloodstain, ***R*** with respect to the cotton, ***R_0_*** is the product of the total absorption and the photon path length, **τ**, as shown in Eq. (3):
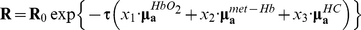
(3)The bold face symbols are considered wavelength dependent. Knowledge of the absorption coefficients of HbO_2_, met-Hb and HC for a large spectral range [5] enables a spectral fit. In blood, hemoglobin is packed in red blood cells of 7 µm diameter, which was accounted for as described in [20]. The spectral fit yields three fit parameters [x_1_, x_2_, x_3_]. These were obtained by minimizing the weighted sum squared error between measured and model estimated reflectance. Since the absorption coefficient is converted for the absorption of whole blood, the fit parameters [x_1_, x_2_, x_3_] represent the fraction of the various hemoglobin derivatives: x_1_ is the fraction of HbO_2_, x_2_ is the fraction of met-Hb, and x_3_ is the fraction of HC. During the fit procedure, the effective mean photon path length **τ** (see Eq (3)) is adjusted simultaneously as a function of the total absorption, while the reduced scattering of the cotton and bloodstain is set to μ_s_′ = 40 mm^−1^ for the entire spectral range. The total blood volume fraction is defined as the sum of x_1_, x_2_ and x_3_. The relative amounts of the various hemoglobin fractions are determined by x_i_/(x_1_+x_2_+x_3_). All steps described above were scripted into Labview code (version 8.6, National Instruments).

### 2.5 Biphasic autoxidation of HbO2 in bloodstains

Following the results of Tsuruga *et al.* [16] the autoxidation process of HbO_2_ is interpreted by initial fast rate and final slow rate as shown in Eq. (2). The fast and slow decay rates are determined at various temperatures from least-squares fits of all measured [HbO_2_] data points to Eq (2).

## Results

### 3.1 OCT measurement of bloodstains on cotton

**Figure 1:**
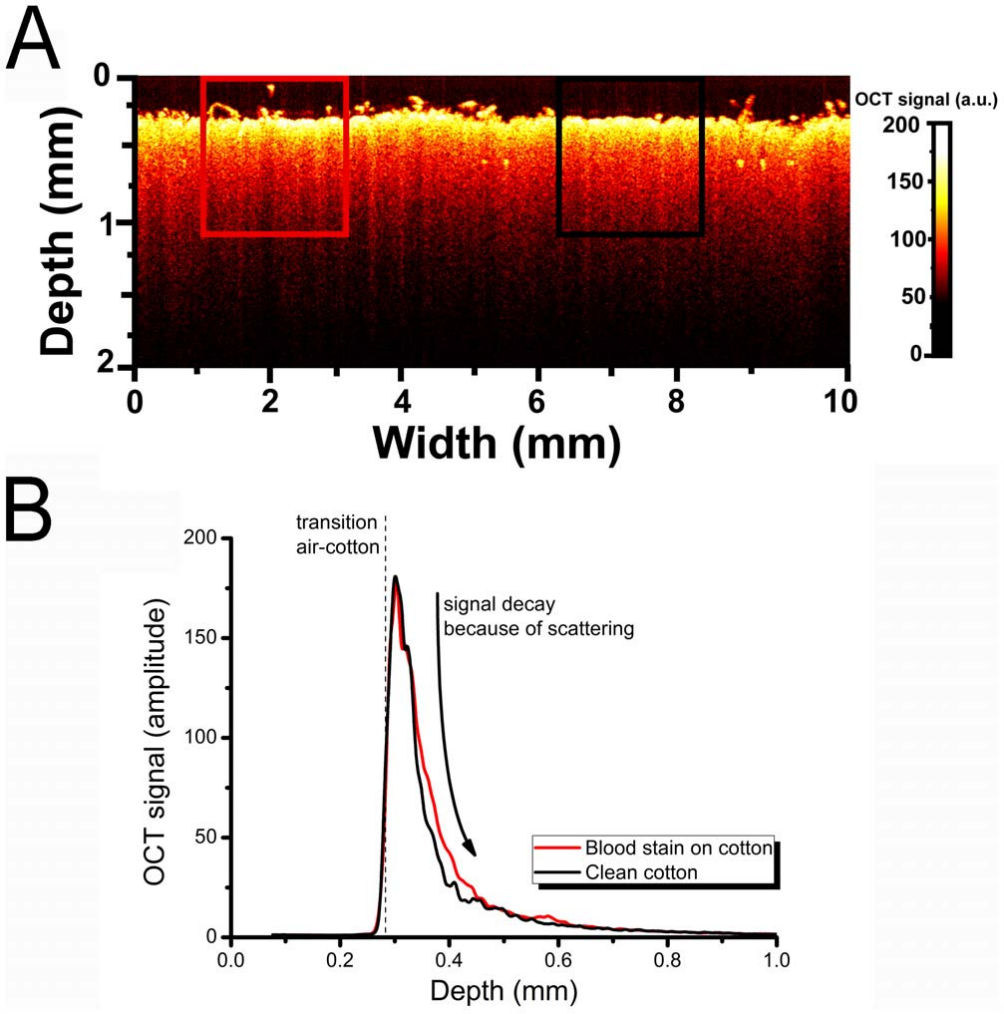
OCT image of bloodstain on cotton. A) Cross-sectional OCT image of cotton fabric with a bloodstain. The left region of interest (red box) shows the position of the bloodstains. The right region of interest (black box) shows a clean spot of cotton fabric. B) Attenuation fits of the OCT signal of bloodstain (red line) and clean cotton (black line).

Figure 1A shows a false color cross-sectional image of cotton fabric. The left side of figure 1A (red box) shows the cotton with a dried bloodstain of 24 hours old; the right hand side the clean cotton (black box). The attenuation of the OCT signal is depicted as the top part of the cotton is colored in white/yellow; the signal attenuates as the light penetrates deeper into the cotton. No differences in structure were observed between the bloodstain and the clean cotton fabric. The two regions of interest for performing attenuations fits are shown in figure 1B. We determined a value of μ_t_ of 16±1 mm^−1^ for the bloodstain and 17±1 mm^−1^ for the clean cotton fabric at 1300 nm.

### 3.2 Bloodstain dilutions

**Figure 2:**
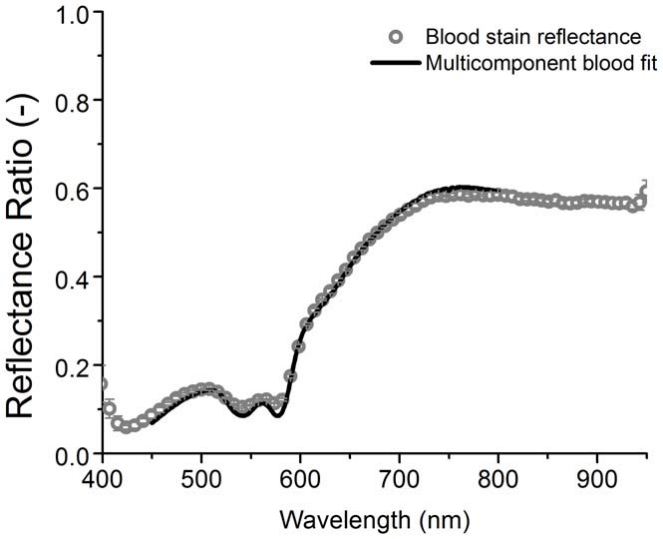
Diffuse reflectance spectrum of a bloodstain. The measured reflectance ratio **R/R_0_** of an undiluted bloodstain (BVF = 1) on cotton fabric measured in the spectral range from 450–800 nm. The gray dots depict the bloodstain reflectance and the solid black line represents the multi-component fit.

**Figure 3:**
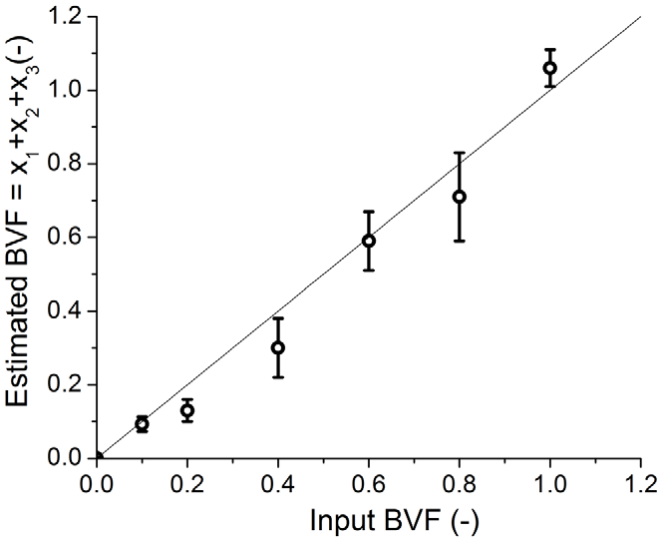
Validation of fit parameters. Optically estimated BVF, based on the spectral fits plotted against input BVF based on the mixing whole blood with PBS for eight various dilutions. The solid line represents the line of unity.

Figure 2 shows the diffuse reflectance ratio **R/R_0_** of an undiluted bloodstain in the spectral range of 450–800 nm. The solid line depicts the multi-component fit based on the three hemoglobin derivatives and the modeled photon path, as written in Eq. (3). The outcome of the fit parameters [x_1_, x_2_, x_3_] represent the fraction of the three hemoglobin derivatives, HbO_2_, met-Hb, and HC, respectively: x_1_ = 0.85±0.04, x_2_ = 0.02±0.01 and x_3_ = 0.19±0.02. The error represents the 95% confidence interval of the fit. The estimated blood volume fraction (BVF) is the sum of the three chromophores, BVF = 1.06±0.05. The estimated blood volume fraction was correctly estimated within 15% for all measurements as evidenced by figure 3. In this figure, the slope of the optically estimated BVF is 0.99±0.05. The correlation between the bloodstain's reflectance and the multi-component fit as shown in figure 2, r^2^ = 0.998.

### 3.3 Autoxidation of HbO2 in bloodstains

**Figure 4:**
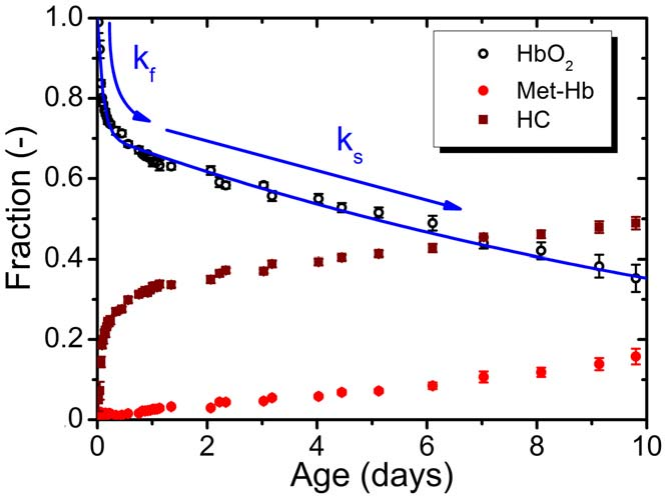
Hemoglobin fractions in ageing bloodstains. Fractions of HbO_2_, met-Hb and HC in bloodstains (n = 3) monitored for ten days. The amount of HbO2 decrease while met-Hb and HC increases after deposition of the bloodstain. Error bars represent the standard deviation. The blue line shows the biphasic oxidation of [HbO_2_].

The outcome of the fit parameters [x_1_, x_2_, x_3_] was used to monitor the ageing bloodstains. To minimize noise on the hemoglobin fraction, we set the sum of the hemoglobin fractions to one for every measurement. Figure 4 plots the relative fractions of the three hemoglobin derivatives, as the average (± the standard deviation) of three bloodstains in the time span of 0–10 days for T = 22°C and humidity is 45±5%. The transition of the HbO_2_ into met-Hb and HC is rapid at first and after ten hours the transition rate changes into a lower rate. The blue line in figure 4 shows the relative oxy-hemoglobin amount fitted by the biphasic oxidation decay Eq. (2). [HbO_2_]_0_ is 1 for a fresh bloodstain since no met-Hb and HC is formed at t = 0. The correlation coefficient between the measured [HbO_2_] and the biphasic autoxidation curve is r^2^ = 0.983.

### 3.4 Temperature

**Figure 5:**
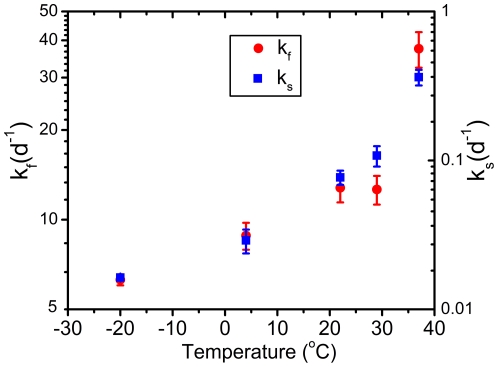
Oxidation parameters vs temperature. Parameters k_f_ and k_s_ as function of temperature. The fast oxidation (k_f_) is plotted on the left axis, and slow oxidation (k_s_) is plotted on the right axis.

Figure 5 shows the biphasic rates k_f_ and k_s_ (± standard deviation) as a function of temperature. The fast rate, k_f_, increases from 6.3 d^−1^ for T = −20°C to 37.5 d^−1^ for 37°C, the slow rate, k_s_, increases from 0.02 d^−1^ for T =  −20°C to 0.36 d^−1^ for 37°C. Also the fraction of the rapid oxidizing hemoglobin, *P* in Eq (2), increases from 0.22 for T = −20°C to 0.39 for 37°C.

### 3.5 Humidity

**Figure 6:**
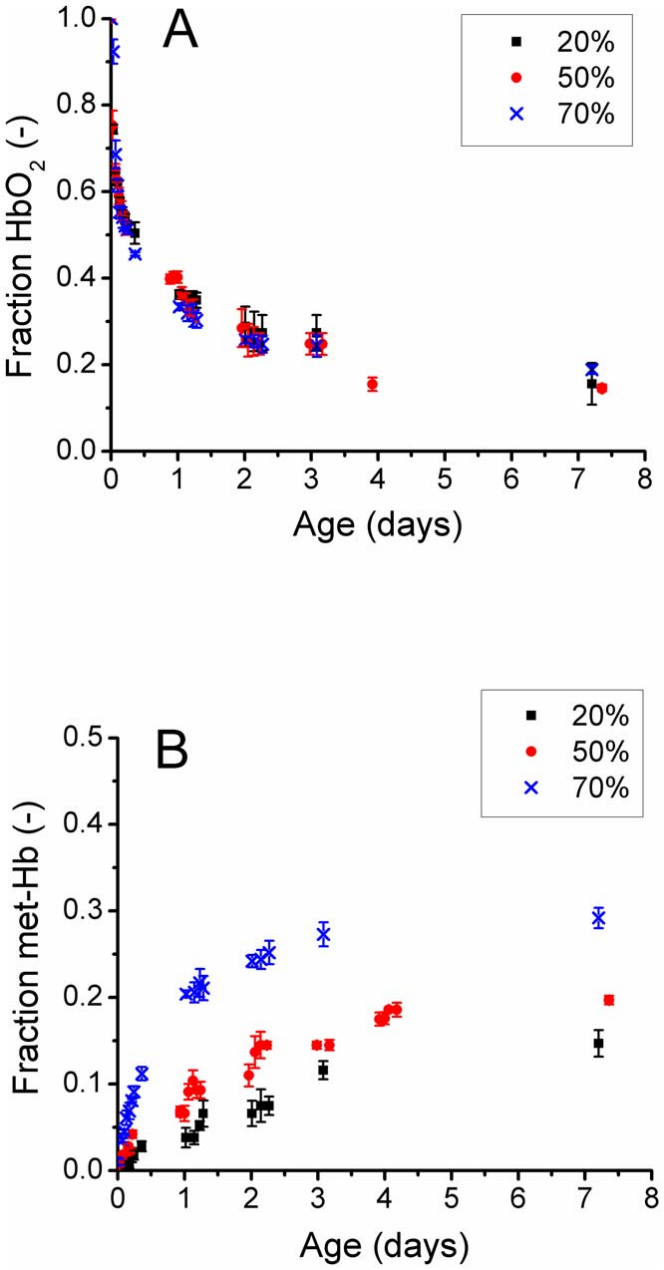
Influence of humidity on hemoglobin fraction. A) HbO_2_ fraction as function of age of the bloodstain for humidity 20%, 50% and 70%. B) met-Hb fractions as function of age of the bloodstain for humidity 20%, 50% and 70%.

Figures 6A and 6B show the influence of humidity on k_1_ and k_2_. Figure 6A shows the fraction of HbO_2_ as a function of age for three values of humidity at T = 37°C. No significant difference is observed in the fraction of HbO_2_ for these three values of humidity.

Figure 6B shows the fraction of met-Hb as a function of age for three values of humidity at T = 37°C. The fraction of met-Hb does depend on humidity; more met-Hb is formed at higher humidity. While the fraction of met-Hb increases as function of humidity and the fraction of HbO_2_ remains constant, the fraction of HC decreases, since the sum of the three hemoglobin derivatives accumulates to 1.

## Discussion

This study shows spectroscopic monitoring of bloodstains to reveal the biphasic oxidation of oxy-hemoglobin in bloodstains. Analysis of the reflectance spectra was used in order to determine the amount of oxy-hemoglobin, met-hemoglobin and hemichrome in a bloodstain. Observations on diluted bloodstains showed that the absolute amounts of hemoglobin derivatives could be determined within 15%. The relative amounts of HbO_2_, met-Hb and HC in bloodstains were followed for ten days at room temperature and could be described by first-order kinetics. We found that the oxidation of HbO_2_ is rapid at first, but decreases after a few hours. These oxidation rates are strongly temperature dependent. The oxidation of HbO_2_ does not depend on humidity, in contrast to the transition of met-Hb into HC which does depend on humidity.

First, the scattering of the substrate with and without the presence of a bloodstain was measured in order to understand the contribution of scattering to the measured signal. We found no significant difference in scattering for clean cotton (17±1 mm^−1^) vs. a bloodstain on cotton (16±1 mm^−1^). This indicates that the scattering of cotton fabric is not influenced by the presence of dried blood, which enables quantitative spectroscopic analysis [7], an observation which was confirmed by validating bloodstain dilutions and shown by a slope of 0.99 in figure 3.

Secondly, the absolute amounts of hemoglobin derivatives were verified. Ideally, amounts of HbO_2_, met-Hb and HC are confirmed individually. However, currently no suitable techniques exist for individual chromophore validation, since most physical properties of HbO_2_, met-Hb, and HC, including molar weight and magnetic properties, are nearly identical. A potential candidate for *in situ* confirmation of the decay of chromophores might be Raman spectroscopy [21], but Raman spectroscopy is hindered by the fluorescing properties of the heme groups [22]. In the present study validation was achieved by diluting whole blood with phosphate-buffered saline and correlating the input dilution to the sum of the determined chromophores. All measurements in this study were performed on samples deposited on white cotton. Samples deposited on non-white substrates require a more sophisticated optical sampling and analysis, with both colored and non-colored background as reference. An alternative to overcome the drawback of background color is the use of a hyper-spectral imaging system similar to what has been used for age determination of bruises [23]. Spectral imaging allows imaging of the bloodstain and its host material and allows both spectral and spatial analysis of the bloodstain. Although this approach is beyond the scope of the present study, it is an interesting topic for future research.

The biphasic oxidation model has a high correlation with the measured [HbO_2_] fraction, r^2^ = 0.983. Yet, around the transition between the fast and slow decay, after 10 h in figure 4, the biphasic model shows small, but consistent deviations from the measured [HbO_2_] fraction. Fitting [HbO2]_t_ with a triphasic model results in a smaller residue, but also requires five instead of three fit parameters. F-test comparison between the bi- and triphasic model over the total time range indicates that the decrease in residue for the triphasic model is smaller than the decrease in degrees of freedom [24]; hence, the biphasic model is preferred. Tsugaru *et al.* and Sugawara *et al.* found that the autoxidation rate of oxy-hemoglobin in aqueous solutions is very much pH dependent [15], [16]. By increasing the level of acidity from pH = 8 to pH = 5, k_s_ increases 10-fold and k_f_ increases 100-fold. In healthy circumstances, blood has pH = 7.4 [25]. Unfortunately, the influence of water evaporation on pH in a bloodstain is not clear. Dehydration also increases the salt concentration, which may influence the autoxidation rate as well [26]. The pH and salt concentration dependence of decay rates may also explain the fact that in our studies we find P = 0.30, while Tsugaru *et al* found P = 0.50, the latter value agreeing well with a priori expectations based on the equal amounts of α– and β–chains in the hemoglobin molecule. Exploration of the pH and temperature dependence on fit parameters P, k_s_ and k_f_ in bloodstains is therefore an important venue for future research. Finally, we observed that the conversion of met-Hb to HC is marked by the inverse biphasic decay of HbO_2_, while the amount of met-Hb increases only slowly. Therefore, the conversion rates of all three hemoglobin derivatives appear more complicated than first-order reaction kinetics and are also topic of future research.

The transition rate of HbO_2_ to met-Hb and HC are very much temperature dependent. We found that the fast oxidation rate of HbO_2_, k_f_, increases 6-fold over the temperature range of −20 to 37°C; the slow oxidation, k_s_, increases 18-fold over the same temperature range. Similar temperature dependencies were found for the transition of met-Hb to HC. The influence of humidity on the transition rate is more complex. We observed that the humidity has no influence on the oxidation of HbO_2_, rate k_1_. However, it does have significant influence on the transition of met-Hb to Hc, rate k_2_. The amount of met-Hb increases as humidity increases. Consequently, the fraction of HC decreases, since the fraction of HbO_2_ is humidity independent and the sum of the three hemoglobin components is set to one. It seems that for the limit humidity→100%, no HC is formed at all, a suggestion which is supported by the observations of Tsuruga *et al.* When measuring HbO_2_ oxidation in an aqueous solution (100% humidity) no HC formation is observed at all [16]. This observation was confirmed in our laboratory (data not shown). For humidity→100%, the rate of met-Hb to HC, k_2_→0. When exploring the opposite limit: humidity→0% it seems that the amount of met-Hb decreases. Possibly for humidity→0%, the transition rate of met-Hb to HC will become larger than the transition of HbO_2_ to met-Hb (k_2_>k_1_) and consequently no met-Hb will be formed.

In conclusion, we have shown that the oxidation of oxy-hemoglobin in bloodstains follows a biphasic decay and that the rates can be described by first-order reaction kinetics. In addition, it was shown that all reaction rates show a positive correlation with temperature and that the transition of met-Hb to HC also depends on humidity. This relationship of the oxidation of bloodstains as a function of temperature and humidity enables the implementation of age determination of bloodstains at a forensic setting, where a wide variety of environmental factors can be encountered.
